# Case of calcinosis cutis associated with Sjogren's syndrome

**DOI:** 10.1002/ccr3.7628

**Published:** 2023-06-26

**Authors:** Keysha Gonzalez‐Ramos, Karishma Ramsubeik, Gurjit Kaeley

**Affiliations:** ^1^ Internal Medicine Department HCA Florida Orange Park Hospital Orange Park Florida USA; ^2^ Rheumatology Department University of Florida – Jacksonville Jacksonville Florida USA

**Keywords:** calcinosis cutis, imaging, Sjogren's syndrome, skin calcification

## Abstract

Calcinosis cutis is a chronic condition involving skin and soft tissue deposition of calcium and phosphate. It is associated with several conditions including idiopathic, iatrogenic, malignant metastasis, calciphylaxis, and connective tissue diseases. The most common connective tissue diseases it is associated with include systemic sclerosis and dermatomyositis. We present a case image of a patient with Sjogren's syndrome and calcinosis cutis and its progression over time. The patient was optimized on her current treatment regimen to prevent further progression. Written informed consent was obtained from the patient to publish this report in accordance with the journal's patient consent policy.

## Graphical abstract

Progressive diffuse calcifications.

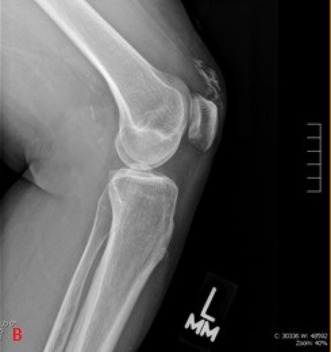

## 1 CASE DESCRIPTION

**FIGURE 1:**
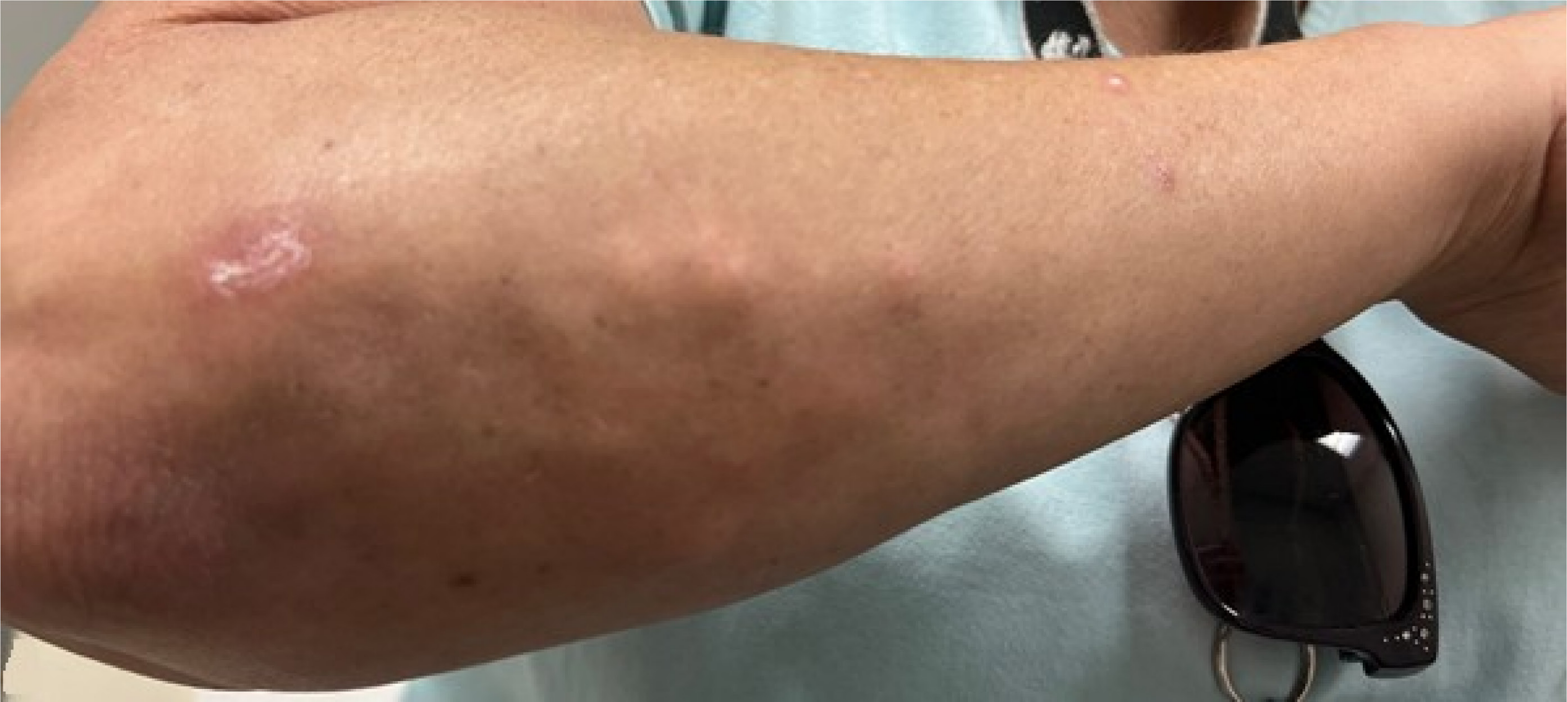
Subcutaneous nodules in the right forearm.

**FIGURE 2:**
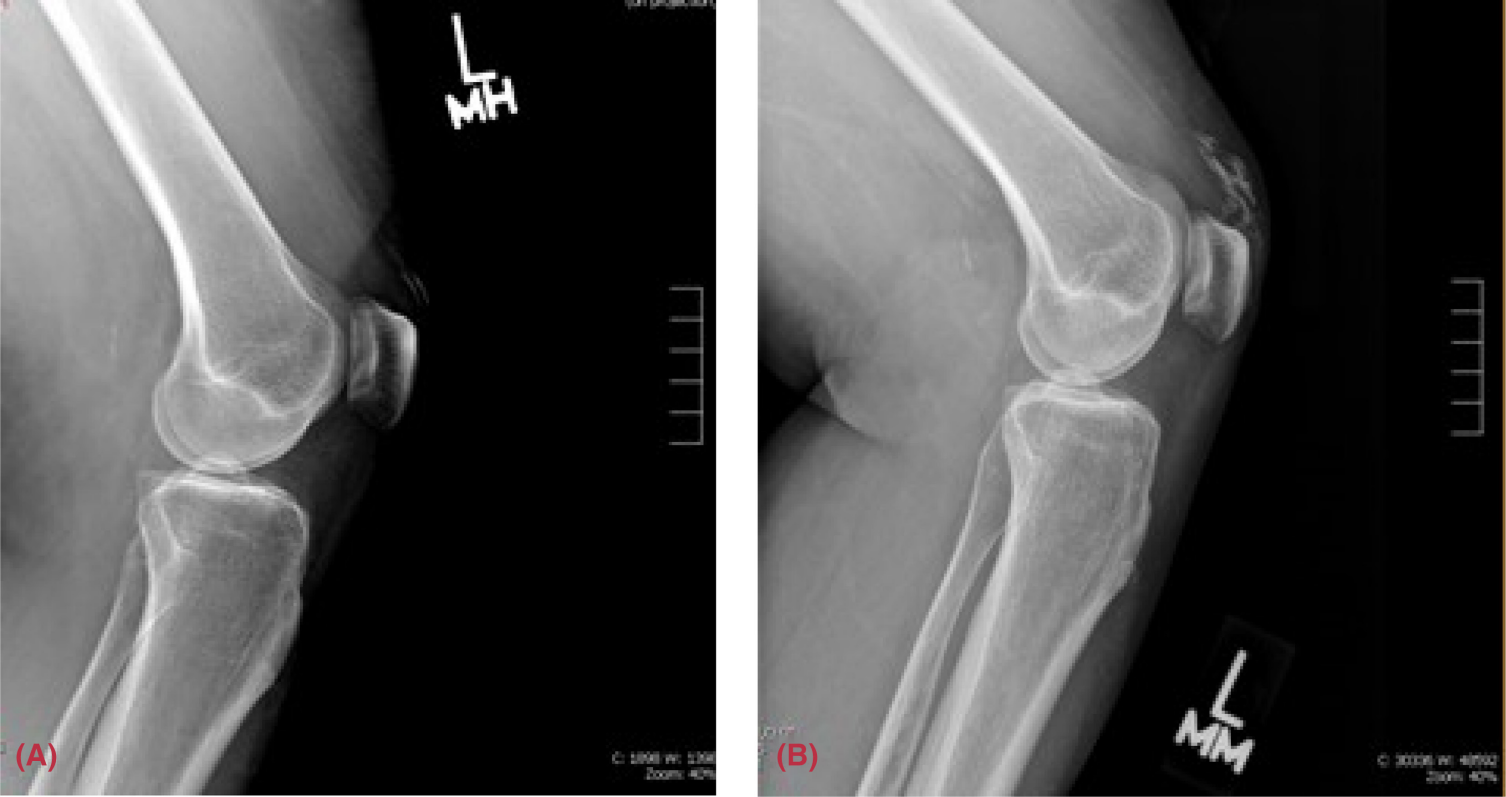
X‐ray of the left knee, lateral view. (A) Mild calcification in the anterior to the distal quadriceps. (B) Progressive diffuse sheet‐like calcifications are anterior to the distal quadriceps 1 year after.

**FIGURE 3:**
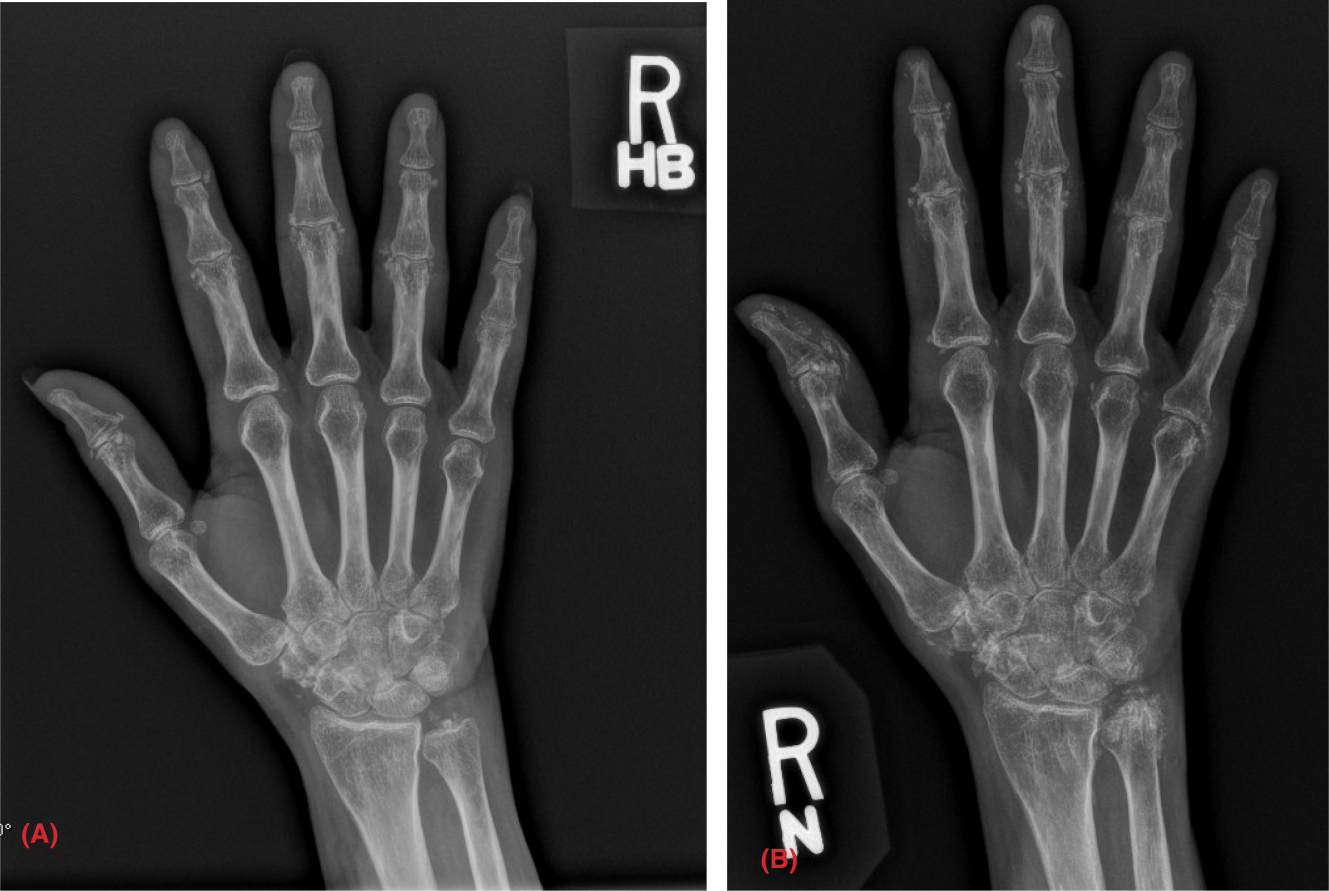
Posteroanterior view of the right hand. (A) Calcifications in PIP joints second to fifth and DIP joints second and fourth. (B) Progressive calcifications in the thumb, PIP joints second to fifth, and DIP joints second to fourth 2 years after.

A 50‐year‐old female with a history of Sjogren's syndrome which manifested by sicca symptoms, positive antinuclear antibody (ANA) titer 1: 320, positive SSA antibody, and a salivary gland lip biopsy that revealed multifocal plasmacytic and lymphocytic sialadenitis with a focus score of 2 greater than 50 lymphocytes present in 4 mm square salivary gland tissue; and calcinosis cutis was seen for follow‐up in the clinic. She is being treated with rituximab, two 1‐g IV infusions separated by 2 weeks administered every 6 months, prednisone 3 mg daily, and diltiazem 240 mg daily. She later developed subcutaneous nodules in her forearms, as displayed in Figure 1, for which she had surgical excision in her left arm, confirming calcinosis cutis on biopsy. She has followed intermittently since then. Radiologic imaging obtained due to persistent pain in the left knee and right hand showed progression of calcifications, as displayed in Figures 2 and 3, respectively.

## 2 CASE DISCUSSION

Calcinosis cutis is a chronic condition with the deposition of calcium salts in the skin and soft tissue. There are five main subtypes: idiopathic, iatrogenic, metastatic, calciphylaxis, and dystrophic. Dystrophic calcification is the most common type of calcinosis cutis and frequently occurs in the setting of underlying connective tissue diseases (CTDs). The most common CTDs include systemic sclerosis and dermatomyositis.^1^, ^2^ This condition can also occur in other CTDs. Three other cases have been reported thus far in association with Sjogren's syndrome.^2^, ^3^ Our patient was started on minocycline 100 mg daily as adjunctive therapy to try to prevent further progression.

## 3 CONCLUSION

Calcinosis cutis can be a feature of several conditions including connective tissue diseases. Among these, the most common are systemic sclerosis and dermatomyositis and rarely Sjogren's syndrome, like our patient. The goals of treatment include stabilization of nodules and avoiding complications like superimposed infection or destruction of a joint.

## AUTHOR CONTRIBUTIONS

**Keysha Gonzalez‐Ramos:** Writing – original draft; writing – review and editing. **Karishma Ramsubeik:** Conceptualization; supervision; validation; visualization; writing – review and editing. **Gurjit Kaeley:** Resources; writing – review and editing.

## FUNDING INFORMATION

The authors did not receive financial support for the research, authorship, and/or publication of this article.

## CONFLICT OF INTEREST STATEMENT

There are no conflicts of interest to declare.

## CONSENT

Written informed consent was obtained from the patient to publish this report in accordance with the journal's patient consent policy.

## Data Availability

Data sharing is not applicable to this article as no new data were created or analyzed in this study.
